# Residual Tensile Strength of the Multi-Impacted Scarf-Repaired Glass Fiber-Reinforced Polymer (GFRP) Composites

**DOI:** 10.3390/ma11122351

**Published:** 2018-11-22

**Authors:** Punita Kumari, Jihui Wang, Saahil

**Affiliations:** 1 School of Material Science and Engineering, Wuhan University of Technology, 122 Luoshi Road, Wuhan 430070, China; punita@whut.edu.cn; 2 School of Automotive Engineering, Wuhan University of Technology, 122 Luoshi Road, Wuhan 430070, China; bitsaahil@gmail.com

**Keywords:** multi-impact test, tensile after impact, residual strength, scarf repair, composites

## Abstract

The effect of multiple/repeated impacts on a repaired composite was investigated using a low-velocity impact test. The composite samples were fabricated through a vacuum resin infusion method (VARI) and repaired by a scarf repair technique. Later, a repeated low-velocity impact test was performed on the original and repaired composites samples. Performance of the multi-impacted repaired and original samples was evaluated and compared by measuring maximum contact force, maximum displacement, maximum time duration, absorbed energy and damage area. Photographs of the post-impacted samples were taken to observe the multi-impact damage progression through visual inspection. The results showed that each repeated impact subjected the samples to more damage. Tensile tests revealed that the scarf repair restored 81.23% strength. It was also observed that the sample obtained the highest damage dent in the low-velocity impact test that failed early during a tensile test and carried the lowest ultimate load.

## 1. Introduction

Adhesively bonded repair technique is preferred over classical mechanical fastening due to stress concentration, weight increment, and structural integrity [1,2]. The goal of repairing is to restore the stiffness and mechanical strength of a damaged composite laminate. Repairing is preferred over replacement because it is one of the most cost-effective techniques, especially when the large panel consists of composites. External patch and internal patch adhesive bonding repairs are two widely used basic methods for composite laminate repair. The main advantages of the internal scarf repair are uniform stress distribution, good load-bearing capacity over the repaired region, smooth or aerodynamic repaired surface, and high stiffness. In addition, the scarf bonded repair is categorized as a permanent repair and generally adopted for sports cars and airplanes [3,4,5,6] where good aerodynamics and smooth surfaces are required. Recently, scarf repair performance against the tensile loading has been tested with over ply placement, scarf ratio variation and optimized fiber orientations performance parameters, which has led to the conclusion that it is capable of restoring 85–90% of damaged strength [7]. Ridha et al. [8] confirmed that the optimized way of scarf repair can restore 95% of strength. In another study, hard patch repairing has shown better results as compared to soft patch repair [9].

In comparison to the numerous advantages, composite repair structure is likely to experience various impact loads and damages. Lightning strike, foreign object damage during maintenance, and hailstone strike are a few incidences of impact load that interact with composites. These incidents cause the matrix crack, fiber breakage, fiber splitting, delamination, and de-bonding, which has led to the failure of composite laminates [8,10,11,12,13,14,15].

Visual inspection technique is one of the primary methods to identify the damage produced after an impact test. Researchers have used visual inspection to evaluate low-velocity damage and determine the damage type [15,16,17,18]. Low-velocity impact on repaired composite laminates can be identified through visual examination [18,19,20]. 

Multi-impact on composite laminates has been studied to understand progressive damage and damage strength degradation [21,22]. Many authors have studied the effect of multi-impact on external patch repair of the composite under specified conditions [11,22,23,24,25]. A few researchers have investigated the residual compressive strength of the repair-impacted composite [11,14,15,26]. Several researchers have done experiments on the residual strength of composite after low-velocity impact [13,20,27]. Nevertheless, to the best of our knowledge, there is no literature available on multi-impact following internal repair composite.

Although the influence of the impact on scarf repair is well studied and investigated [10,12,13,28], repercussions of multi-impact on scarf-repaired glass fiber-reinforced polymer (GFRP) laminates has not yet been evaluated with much consideration. In addition to this, the damage mechanism and the effects of scarf repair under multiple impacts is not very clear. Moreover, there has been no investigation or work done on the residual tensile strength after repeated impacts on scarf-repaired GFRP. Hence, in the present study, the effect of repeated impacts on scarf-repaired GFRP is tested.

Furthermore, the impact results are compared in terms of maximum contact force, time duration, maximum displacement, absorbed energy, damage depth, and damaged area. The tensile test has been conducted on the impacted repaired GFRP to acquire the residual tensile strength after the impact test. Both the impacted repair and original samples were investigated through visual inspection to detect the damage type. Repaired and original GFRP samples were subjected to multi-impacts and residual tensile strength was tested through tensile after impact (TAI). A relationship between the residual strength of the sample after impact repair and strength of the original sample was established on a comparable basis.

## 2. Experimental Procedure

### 2.1. Material Preparation

#### 2.1.1. Manufacturing of Composite

**Figure 1:**
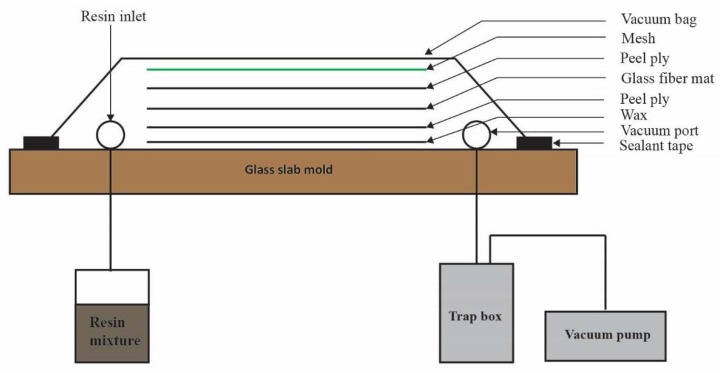
Schematic diagram of VARI.

Unidirectional glass fiber and vinyl ester resin were used to manufacture the parent and scarf patch composite laminates. A glass table was selected as mold and manufacturing of the GFRP was done on it. E-glass fiber fabric with 430 GSM (gram square meter) was purchased from the Chongqing Polycomp International Corporation, Chongqing, China. Before being used, it was cut and dehydrated in an oven at 40–50 °C for 5–7 min to remove any moisture present in the fabric. The 16 layers of glass fiber fabric were stacked together following [0/90/90/0]_2s_ stacking sequence and fabricating through VARI (vacuum resin infusion) technique [29] and details are shown in Figure 1. The weight ratio of glass fiber to resin mixture (60:40) was selected according to the manufacturer’s suggestion. The resin mixture was prepared using vinyl ester (sw905-2 supplied from SWANCOR IND. CO., LTD., Shanghai, China), hardener (methyl ethyl ketone peroxide (NOROX KP-925H) from United Initiators, Stockholm, Sweden) and initiator (accelerator cobalt salt 1305 from SWANCOR IND. CO., LTD., Shanghai, China). The same amount (0.5% weight of the resin) was used for both. The resin transfer took 45–50 min for impregnation through all 16 layers of fibers fabric. After the insertion of resin impregnated glass, the fiber system was left to cure for 24 h at ambient pressure and temperature. The uniform thickness of 4.8 mm was obtained after curing with 300 × 100 mm dimension [6,13], marked on the GFRP and 33 samples were trimmed by water wafering saw from the composite plate, 15 for original and 15 for repairing procedure. Water absorption was removed during trimming process through drying in an oven at 40–50 °C for 10–12 min. The dried samples were wiped with cotton to remove any residual water or dust particle. Samples used for impact testing were categorized into original samples (group 1) and repaired samples (group 2) as shown in Table 1. All the impact tests were performed in triplicate.

#### 2.1.2. Repair Technique

**Figure 2:**
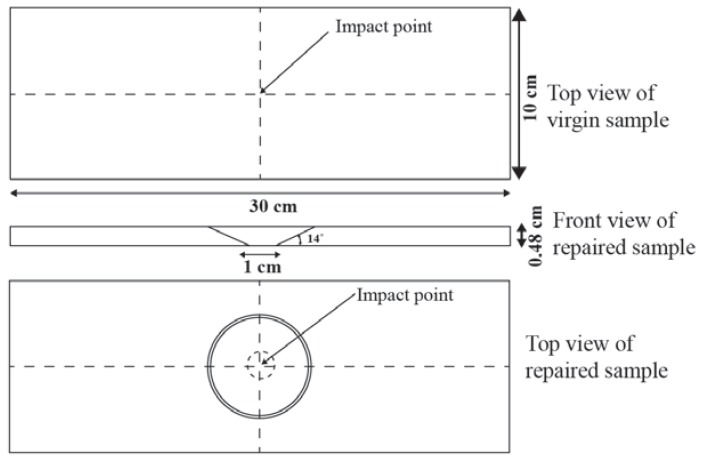
Dimensions of the repaired and original composite samples.

**Figure 3:**
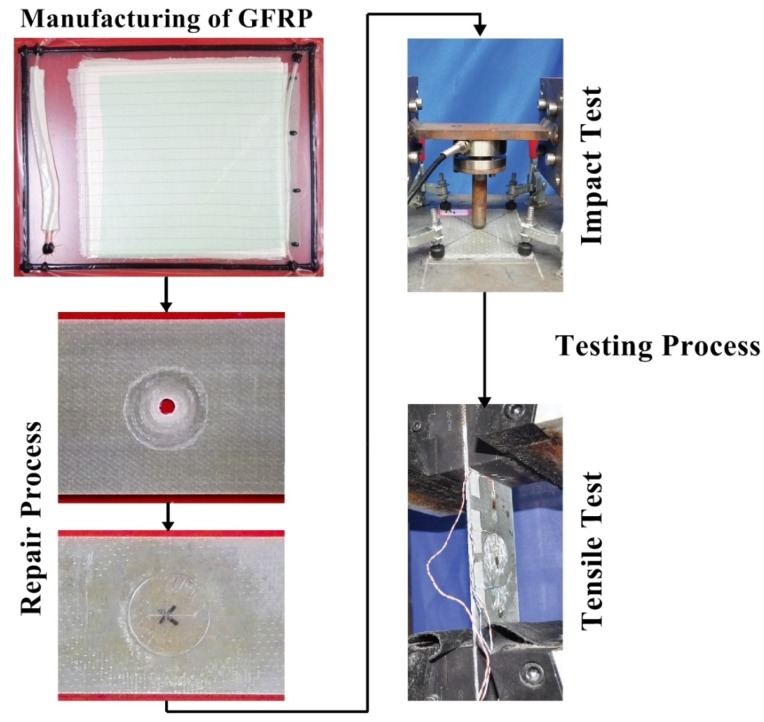
Manufacturing, repairing and testing procedure.

After cleaning, trimmed samples (15) were marked with 10 mm diameter hole at the center (Figure 2) of the GFRP samples and drill operation was performed using CNC (computer numerical control) machine. The dust on the samples was cleaned using cotton that was soaked in acetone. The 14° scarf angle was marked on the samples and scarfing was done carefully using the rotatory sad paper tool. The scarfed region was polished for a good bonding strength. For each scarfed sample, the patch was cut out from GFRP containing same architecture and thickness as in parent laminate according to the scarfed size and scarfing was done same as the parent laminate. Before doing repair operation, the surface of the patch and scarfed samples were wiped with acetone with help of cotton to avoid any contamination such as oil, grease, dust particle and residual as per American society for testing and materials (ASTM) [30]. The patch was applied to the scarfed region of the composite sample. The patch fully covered the scarfed region, to get the best results of bonding between the patch and scarfed samples. Resin mixture (vinyl ester, hardener, and initiator) was prepared with the same weight proportion as of parent manufacture. The mixture was kept in a pressure vassal for 3–4 min to avoid the formation of bubbles during the mixing process. A small amount of resin mixture was applied to the scarfed region with the help of the brush and covered with the patch. While putting the patch, the patch axis angle was kept same as parent laminate, and to remove the excess resin, little weight was applied on it and left for 72 h to cure. After that, the weight was removed from the samples and repaired samples were left for one week for post curing to dry and acquire good bonding strength. The whole repair process was performed at room temperature. The procedure for manufacturing, repairing and testing is explained in Figure 3.

### 2.2. Testing Procedure

#### 2.2.1. Drop Weight Impact Test

**Figure 4:**
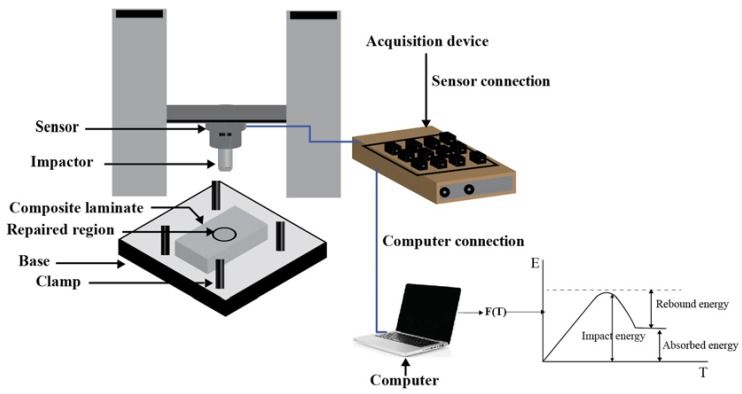
Schematic diagram of drop weight impact machine.

After performing the repair technique, both original and repaired composite samples were marked and subjected to the low-velocity impact test using drop weight-impact machine (Figure 4). A dart of 8 mm radius, 8.9 kg weight and hemispherical geometry of the impact was used in this work. The extended part of dart was fitted with ball wearing that allowed it to move freely on the guide rail. A force sensor was mounted on the impact dart and connected to the acquisition device to record force-time history for each impact. The required initial velocity was obtained after some modifications in impact height. Samples were clamped to the test bench as per ASTM D7136 [31], it did not allow the sample to move due to vibration produced during the strike. Since in this work, visual inspection was preferred among all non-destructive techniques. Few samples with a dimension of 150 × 100 mm were cut and tested with various impact energies according to ASTM D7136 to obtain the visual damage at the impacted surface. For this particular study, impact energy of 15 J was found suitable for multi-impact in both original and repaired sample cases. The impact dart was fixed at 171.97 mm height from the GFRP composite sample to achieve impact energy of 15 J. The impact dart was released from the height and hit to the samples, while collision kinetic energy was transferred to the sample that caused damage. For each collision, data was recorded by the acquisition device and stored in the computer. After the first impact, the damage depth was measured through a dial indicator. Later, the impacted samples were unclamped and removed carefully from the fixture to take images of the damaged area than another sample was clamped for the second impact and impact dart was subjected twice at the center. After this, the damage depth of the second impacted sample was measured. The same procedure was followed for the third and fourth impact. After the impact tests, samples were removed from the test fixture and preserved for post impact visual inspection and photography was done using a digital camera (DSC-RX10 II, Sony Inc., Tokyo, Japan). For further analyses, damage area measurement of impacted samples was recorded, and different damage types were shown through the photographic images. The equations used in impact energy and absorb energy calculation is as:

(1) E=mgH

(2) $$E=\frac{mv_{i}^{2}}{2}$$

(3) $$v\left(t\right)=v_{i}+gt-\int_{0}^{t}\frac{F\left(t\right)}{m}dt$$

(4) $$\delta \left(t\right)=\delta_{i}+v_{i}t+\frac{gt^{2}}{2}-\int_{0}^{t}\left(\int_{0}^{t}\frac{F\left(t\right)}{m}dt\right)dt$$

(5) $$E_{a}\left(t\right)=\frac{m\left(v_{i}^{2}-v{\left(t\right)}^{2}\right)}{2}+mg\delta \left(t\right)$$

where *E* = Impact energy (J), *H* = Impact height (m), *m* = mass of the impact ball (kg), *g* = gravitational acceleration (9.81 m/s^2^), $v_{i}$ = Impact velocity (m/s), v(t) = Impact ball velocity at time t, δ(t) = Impact ball displacement at time t, F(t) = Contact force at time t, and $E_{a}\left(t\right)$ = Absorbed energy by the samples after impact test.

#### 2.2.2. Tensile Test

After the damage area measurement and visualization of the repeated impact damage to the repaired and original composite sample, the residual strength of multi-impacted samples tested under tensile loading (Figure 3). The prepared sample was fitted to the universal testing machine (UTM) jaw and set crosshead speed (2 mm/min) [6]. After achieving the ultimate load, the sample was removed from the UTM jaw and photos were taken to visualize the damage mechanism. Three samples were tested, and the mean value was used in calculation and graph plotting. The connection of the data acquisition device, sensor and computer is illustrated in Figure 4. 

## 3. Result and Discussion

### 3.1. Impact Test

#### 3.1.1. Force—Time Response

**Figure 5:**
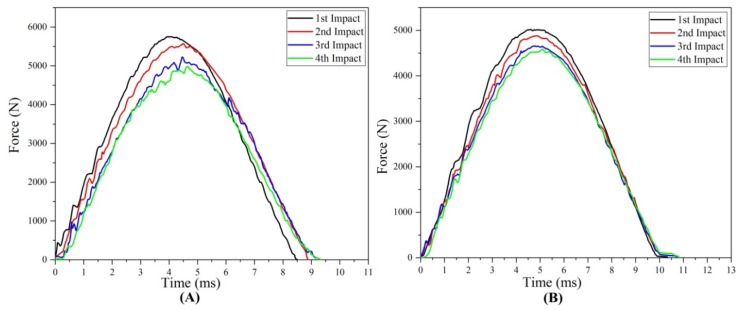
Force-time graph of (**A**) original and (**B**) repaired composite impacted composite samples.

**Figure 6:**
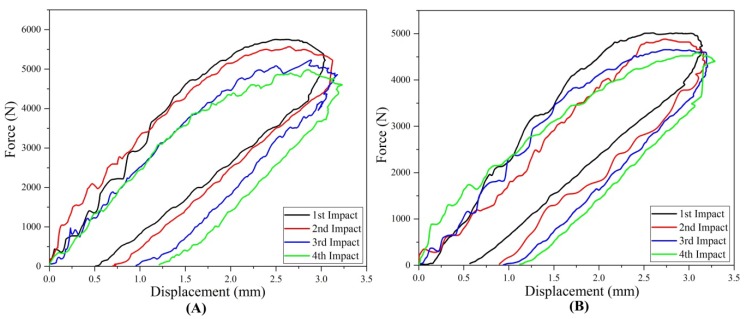
Force-displacement graph of (**A**) original and (**B**) repaired impacted composite samples.

**Figure 7:**
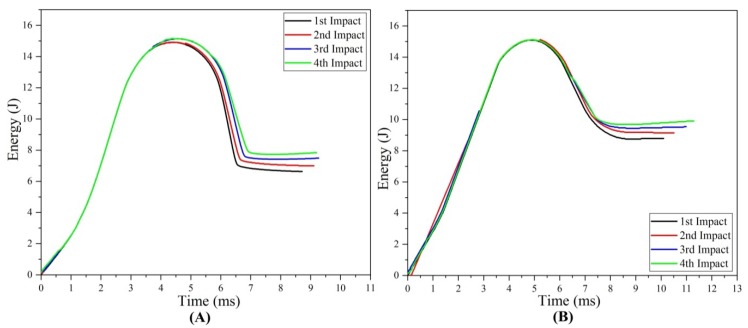
Absorbed energy- time curve of (**A**) original (**B**) repaired impacted samples.

**Figure 8:**
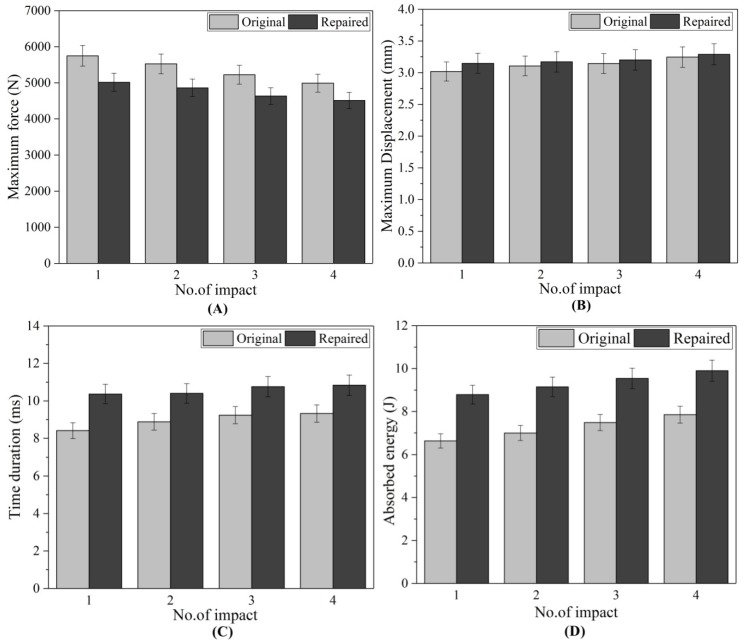

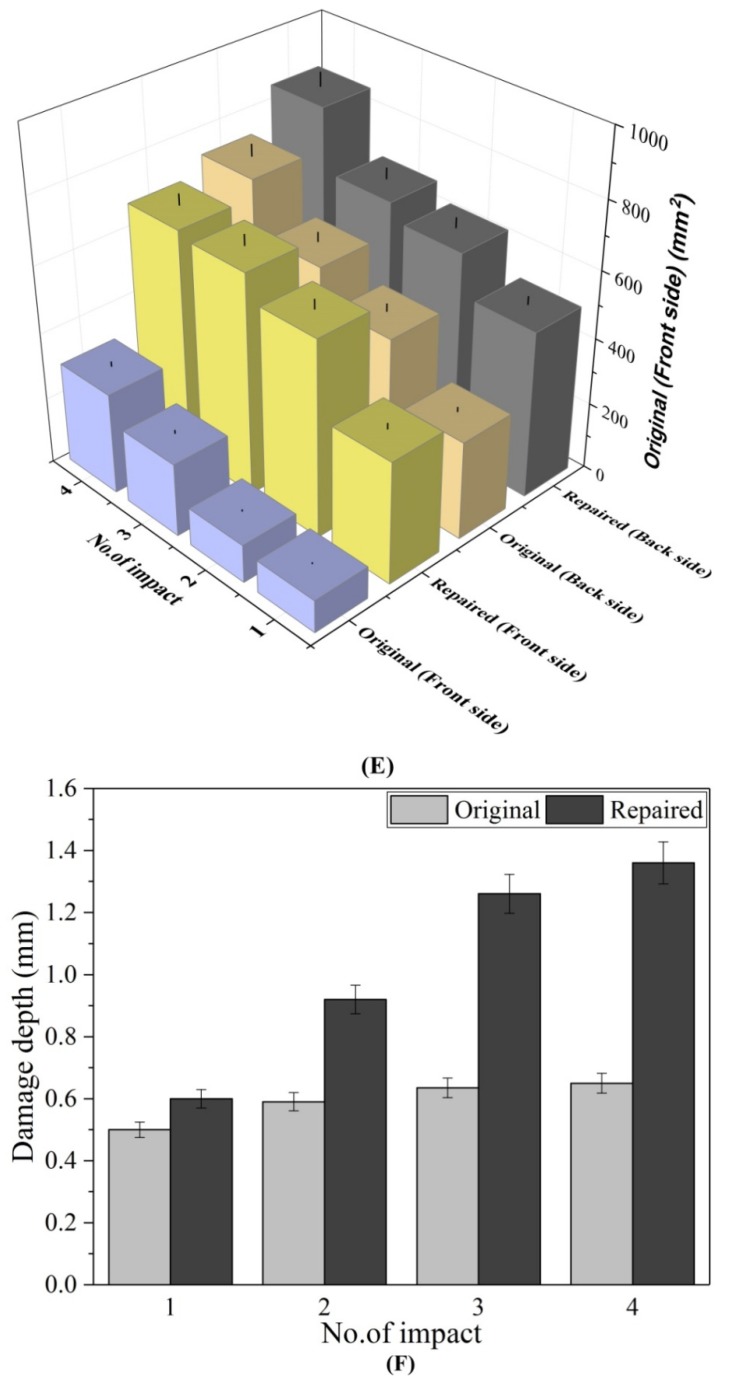
(**A**) Maximum contact force (**B**) maximum displacement (**C**) Time duration (**D**) energy absorption increment (**E**) damaged area (**F**) damaged depth obtained by composite original and repaired samples.

**Figure 9:**
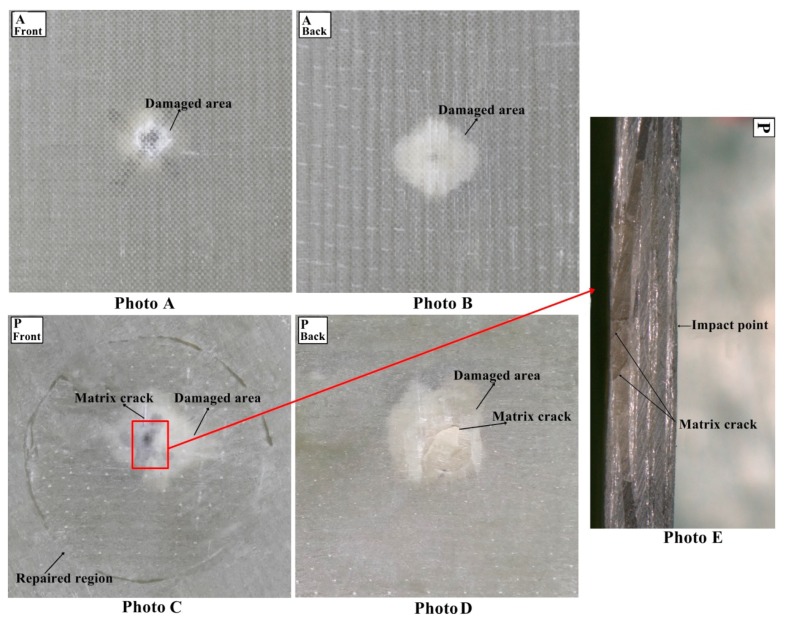
(**A**) Front face of original (**B**) back face of original (**C**) front face of repaired (**D**) back face of repaired impacted (**E**) cross-sectional view of the repaired composite sample after first impact.

**Figure 10:**
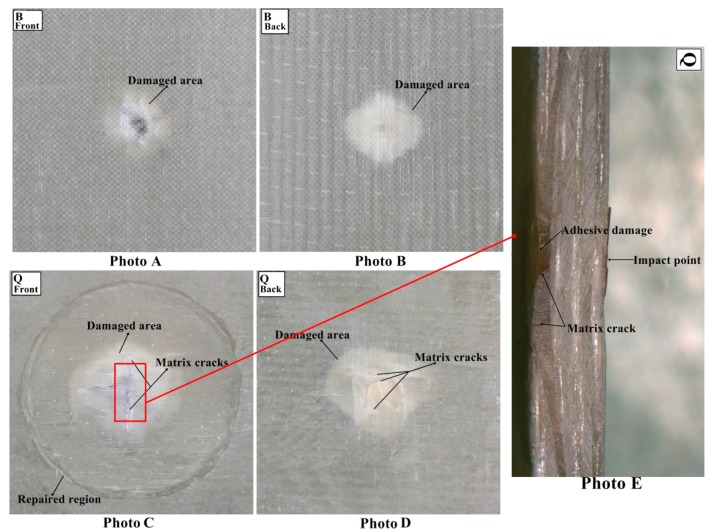
(**A**) Front face of original (**B**) back face of original (**C**) front face of repaired (**D**) back face of repaired impacted (**E**) cross-sectional view of the repaired composite sample after second impact.

**Figure 11:**
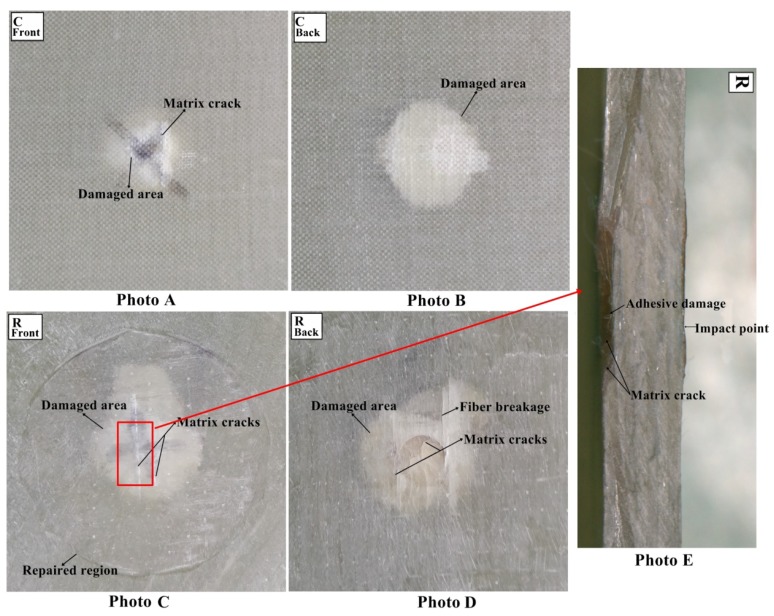
(**A**) Front face of original (**B**) back face of original (**C**) front face of repaired (**D**) back face of repaired impacted (**E**) cross-sectional view of repaired composite sample after third impact.

**Figure 12:**
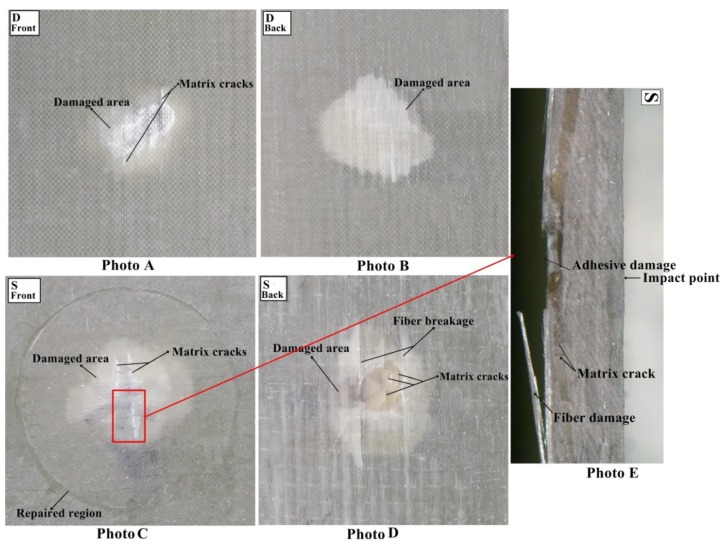
(**A**) Front face of original (**B**) back face of original (**C**) front face of repaired (**D**) back face of repaired impacted (**E**) cross-sectional view of the repaired composite sample after fourth impact.

The results of contact force-time, force-displacement, and absorbed energy-time curves for repaired and original test samples for each impact are shown in Figure 5, Figure 6 and Figure 7. Figure 8A–F represents the comparison study of maximum contact force, maximum displacement, time duration, energy absorption, damaged area and damaged depth acquired by composites original and repaired samples respectively. Figure 9, Figure 10, Figure 11 and Figure 12 shows the visual inspection result of original and repaired samples after multi-impacts. In Figure 5, the curve of original coupons showed almost the same response against impact; the curve raised with time and after obtaining peak force it started to decrease with time. The shape of the curve was an inverted “V”. This showed that contact force was maximum at first impact and start reducing with several impacts. The contact force depicted the strength capability of the composites during low-velocity impact. The composite sample type A obtained the highest force with the intended lowest absorbed energy, as displayed in Figure 9A. It was revealed that the contact force was inversely proportional to the damage induced in samples after the impact test. Original composite samples attained maximum peak force compared to the repaired composite for each impact as shown in Figure 5. Contact force of the original and repaired composite can be ranked from higher to lower as follows: type A > type B > type C > type D and type P > type Q > type R > type S, respectively.

The contact force-time duration upon first impact was lowest and increased with the number of impacts for both repaired and original samples. At fourth impact, the time duration was highest. From this, it can be explained that if the time duration is higher, the composite obtains greater impairment. If the time duration is high it means the contact between impact dart and sample will be greater, which might have allowed the dart to penetrate more into the composite sample, hence more damage was induced. Time duration was directly proportional to the damage induced to the samples as reported by the previous study [11]. However, at the fourth impact time, duration was found to be maximum for repaired and original samples; repaired composite samples exhibited more time duration than the original composite laminates (Figure 5). Time duration during impact tests of the original and repaired samples can be ranked as following order: type A < type B < type C < type D and type P < type Q < type R < type S, respectively.

##### Comparative Study

A comparative study between multi-impacted original and repaired samples was conducted based on maximum force and time duration. The percentage of maximum force variation with respect to original samples at first, second, third and fourth impacts was calculated as 12.74, 11.98, 11.33 and 9.60% respectively. The decreased percentage per impact showed that the force value decreased with the high number of impacts. However, the variation in percentage of observed time duration with respect to original samples at the first, second, third and fourth impact was −23.26, −17.11, −16.40 and −16.17%, respectively. The increment in the time duration with several impacts was observed.

#### 3.1.2. Force-Displacement Response

Force and displacement curve of repaired and original composite samples for each impact is presented in Figure 6. For each impact, the contact force started from zero and increased with the displacement after reaching the peak force and maximum displacement started to decrease gradually. The closed curve of force verse displacement was obtained for original and repaired composite samples under multi-impact test. The samples obtained more displacement representing the less stiff nature of the composite and permanent deformation [24]. In both repaired and original cases, fourth impact on the composite demonstrated the highest value of displacement. Therefore, it was found that the fourth impacted coupons achieved the highest permanent deformation compared to all impacts as shown in Figure 8F. The maximum displacements obtained by the original and repaired coupon can be ranked as follows: type D > type C > type B > type A and type S > type R > type Q > type P, respectively.

##### Comparative Study

Comparative study of maximum displacement achieved by the multi-impacted original and repaired samples was carried out. The percentage of the maximum displacement variation with respect to the original samples at first, second, third and fourth impacts were calculated as −4.31, −2.25, −1.92 and −1.23%, respectively. The increasing order of the displacement per impact demonstrated the energy absorption.

#### 3.1.3. Absorbed Energy

Absorbed energy can be defined as the difference between rebound energy and impact energy. When the impact dart was released from a height, the initial and potential energies changed into kinetic energy. When the impact dart hit the composite sample, it absorbs a small amount of energy and the remaining energy stored back in the dart is determined as “rebound energy”. The energy absorbed by the composite samples resulted in damages such as delamination, de-bonding, cracks, and fiber failure. This meant that the composite samples that absorbed more energy got more deformed and incurred more damage. With the addition of multi-impact, the absorbed energy increased, which was aligned with the previous study [21]. The energy absorption trends in original and repaired samples can be listed as: type D > type C > type B > type A and type S > type R > type Q > type P, respectively (Figure 7).

##### Comparative Study

The comparative energy absorption of the multi-impacted original and repaired samples was studied. The percentage of absorb energy with respect to original sample at first, second, third and fourth impact was calculated as −32.42, −30.62, −27.54 and −26.27%, respectively. The increment percentage with the number of impacts revealed that the energy absorption during the fourth impact was highest in both original and repaired samples.

#### 3.1.4. Damage Analysis

The multi-impacted composite sample was investigated through visual inspection. It was noticed that front faces of all composites got more damage than back faces (Figure 9, Figure 10, Figure 11 and Figure 12). There was no major damage except for local matrix failure in the original sample, whereas matrix cracks on the patch and parent of repaired composite appeared after the first impact loading, as shown in Figure 9. At second impact, multiple cracks were formed on the back face of the repaired samples, while there was no major damage incurred to the front and back of the original sample. The cracks that developed upon the first impact were enlarged upon second impact in the repaired sample and spread towards patch edge sides (Figure 10). At the third impact, a small matrix crack was observed on the front face of the impacted original sample and local matrix with increased damaged area experienced a trough as shown in the visually inspected images of Figure 11A,B. Cracks at the repaired hole area were raised for composite samples; at the front face, matrix cracks expanded, whereas multiple matrix cracks around the repaired hole appeared including little fiber breakage at the damaged hole edge area along with partial delamination (Figure 11C,D). Upon fourth impact, multiple cracks were formed at the front face of the original multi-impacted samples, and on the back face of the local matrix, damage increased. On the back face of the repaired composite, fiber failure occurred along with multiple cracks (Figure 12D). However, visual inspection did not provide internal failure among fiber layers, which can be determined through other techniques, such as micrograph.

The value of maximum peak force of each impact for the original and repaired samples is plotted in Figure 8A. The bar graph shows that the original samples achieved higher impact contact force than repaired samples and it was reduced with the number of impacts. Peak contact force was highest for first impacted original sample and lowest for the four-times impacted original sample. The same observation was noticed for impacted repair sample. However, the obtained maximum force was significantly lesser than the original sample. The peak force order can be given as: A > B > C > D and P > Q > R > S for original and repaired samples, as is clear from Figure 8. The maximum displacement value of the repaired sample was higher than the original samples for each impact that revealed the brittle behavior of the repaired composites sample. These findings are in close agreement with the results reported by other researchers [24] (Figure 8). The addition of the matrix to the repaired GFRP laminate made it brittle and less stiff than the original sample as shown in damaged visual inspection photos (Figure 9, Figure 10, Figure 11 and Figure 12). The maximum displacement data obtained from the fourth impact test was higher than all impact tests, thus the samples obtained more damage. The maximum displacement order for original and repair sample was observed as: D > C > B > A and S > R > Q > P, respectively. The contact time duration for original and repair samples can be given as: D > C > B > A and S > R > Q > P, respectively. Figure 8C also showed that the repaired samples got more penetration by impact dart which might have been a result of more contact time and more damage (studied from visual inspection impacted photos). From Figure 8D, it was obvious that energy absorbed by the original sample was less than the repaired sample. Both cases demonstrated that four-times impacted samples absorbed more energy and attained more damage. In all multi-impact cases, the repaired sample absorbed more energy in the form of matrix crack, fiber breakage, and delamination, which can be observed in damaged inspected photographs. From Figure 8D, the order of energy absorbed by original and repair sample can be ranked out as D > C > B > A and S > R > Q > P, respectively. The performances of the original and repaired samples under low-velocity impact are mentioned in Table 2. The bar chart between the number of impacts and the damaged area of the front- and back-face samples is demonstrated in Figure 8E. The results showed that the damaged area increased with several impacts. It was also observed that the repaired sample occupied more damage than the original sample. The backside of each repaired and original sample attributed more damage than the front, can be observed during a visual inspection of sample. For both samples, front and back faces, the damaged area can be ranked from higher to lower in the following order: D > C > B > A and S > R > Q > P, respectively. The damage depth was measured after each impact sample and the order was D > C > B > A and S > R > Q > P for original and repair sample, respectively.

### 3.2. Post-Tensile Test

**Figure 13:**
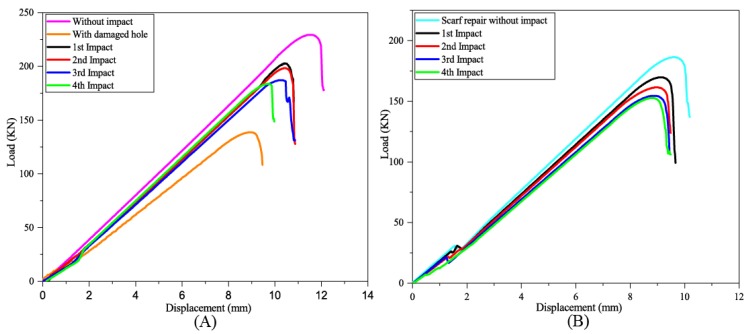
Residual tensile test result of multi-impacted (**A**) original and (**B**) repaired samples.

**Figure 14:**
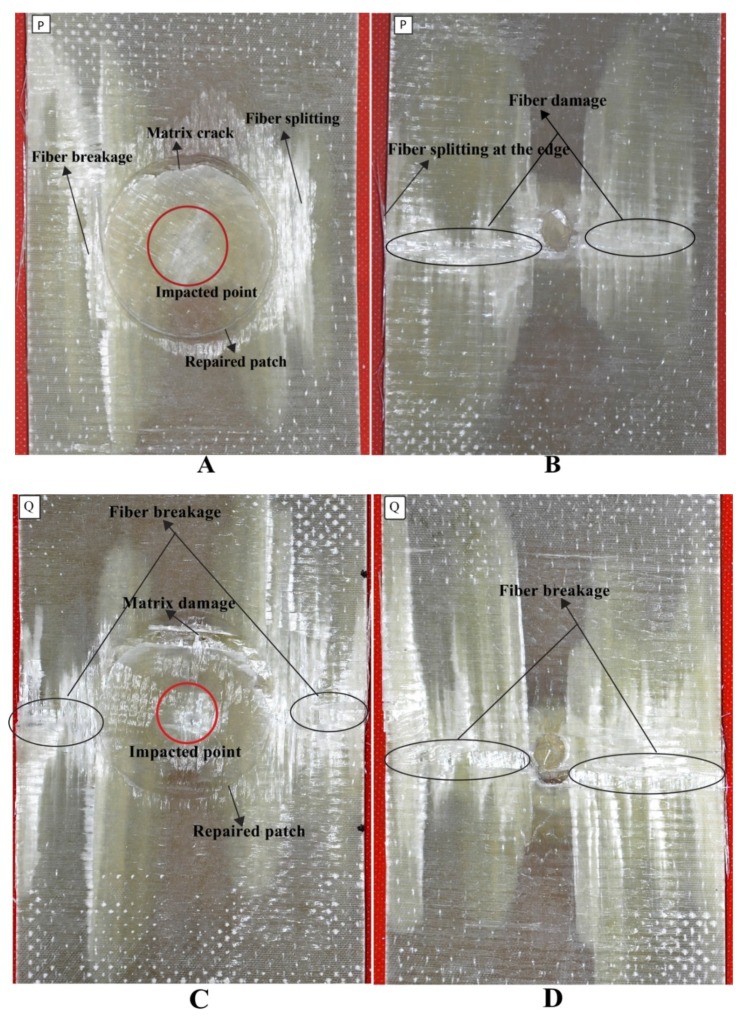

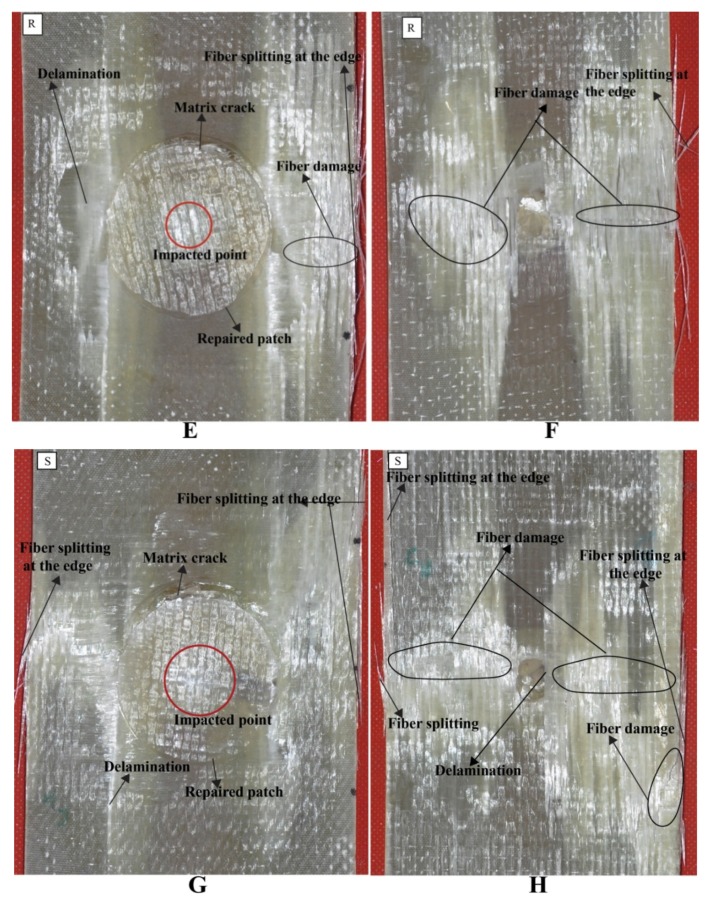
(**A**) Front face of repaired GFRP after first impact (B) Back face of repaired GFRP after first impact (**C**) Front face of repaired GFRP after second impact (**D**) Back face of repaired GFRP after second impact (**E**) Front face of repaired GFRP after third impact (**F**) Back face of repaired GFRP after third impact (**G**) Front face of repaired GFRP after fourth impact (**H**) Back face of repaired GFRP after fourth impact after tensile test.

**Figure 15:**
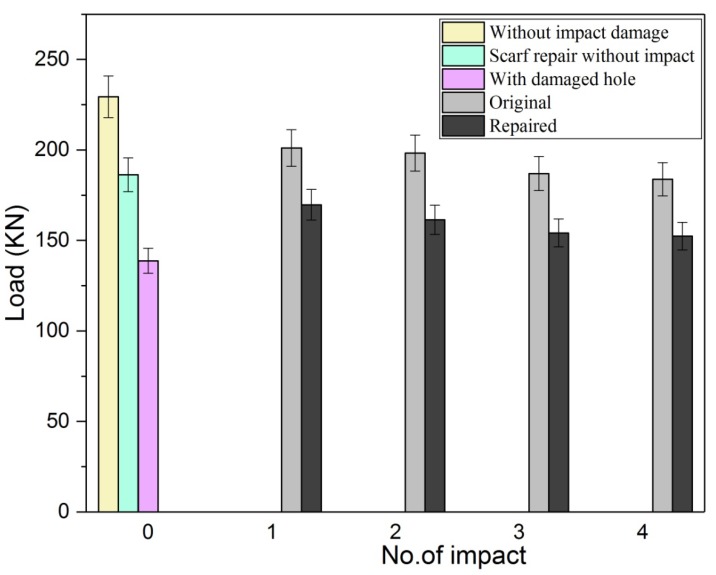
Tensile maximum load verse number original, repaired, damaged samples.

The residual strength of the multi-impact, for both original and repair composite samples was tested through tensile test, and the results are shown in Figure 13. The tensile load-carrying capacity of the impacted samples was compared with the normal samples (without impact strength). Samples without impact and damaged samples exhibited the highest load as expected during tensile loading. The tensile load-carrying order of original sample and scarf samples can be depicted as follows: Sample without impact > A > B > C > D > damaged sample and scarf sample without impact > P > Q > R > S, respectively. In both cases, the fourth impacted sample displayed the least load-carrying capacity. In Figure 14, the post-tensile response of repaired multi-impacted composite samples is illustrated. In the case of all repaired samples, the adhesive bond attachment between parent and the patch existed even after the tensile test except little detachment was observed on the patch edge towards the tensile direction. The stress concentration was generated at the scarfed repair region. As expected, the plane composite sample without being subjected to any impact depicted high tensile load and the least damage, whereas the four-times impacted coupon displayed the worst tensile response among all composite samples. Repaired composite samples demonstrated less tensile load compared to the original composite, as mentioned in Figure 15.

With the number of impacts, the tensile load-carrying capacity of both repaired and original composite was reduced. Scarf repair samples without impact were able to recover 81.23% of the strength as shown in the curve (load vs. displacement) of the tensile test (Figure 13). Percentage load variation of repaired samples with respect to the original sample at first, second, third and fourth impacts were calculated as 19.56, 18.6, 17.55 and 17.08%, respectively as mentioned in Table 3. However, the percentage load variation of original samples with respect to original sample without impact for first, second, third and fourth impact was computed as 9.32, 13.55, 18.48 and 19.89%, respectively. While the tensile load percentage variation of repaired samples with respect to the repair without impact sample at first, second, third and fourth impacts were found to be 10.27, 13.38, 17.26 and 18.23%, respectively.

The main reason for conducting the tensile test on original and repaired composite was to demonstrate residual tensile strength after the impact test. During the tensile test, a crack sound was heard but there was no sign of damage or crack detected through visual inspection, while the graph kept going after a little fluctuation in the curve and dropped after achieving the ultimate load. It implies that internal damage might have occurred. It was also noticed that the crack sound came only for repaired samples. The tensile test result of the P sample revealed that the fracture occurred at the scarf-repaired zone, fiber breakage, and delamination, as can be seen in Figure 15A. A small amount of matrix failure, fiber fracture with splitting was noticed on the backside. The fiber splitting occurred at the edge of the composite sample. Sample Q demonstrated more fracture damage than sample P. At second impact, the center part zone obtained more damage and failed before the P coupon; fiber damage was identified on the front face while on the back-face fiber opening and disintegration occurred. Meanwhile the patch remained stuck to the parent laminate. Delamination and fiber splitting was also developed no the front face of R composite samples after three multi-impact and tensile tests. Delamination occurred around the scarf periphery due to high stress concentration under tensile loading. The third impact developed internal damage to the repaired coupon. When the tensile load was applied to it, the fiber was pulled out and splitting came out from the edge of the sample as presented in Figure 15F. The matrix failure occurred at the repaired area but the patch remained attached to the parent laminate after complete fiber failure under tensile loading condition. Due to highest energy absorption during impact test, the S coupon failed early during the tensile test (Figure 14B). It obtained more damage among all repaired coupons as displayed in Figure 15G,H. Delamination, fiber damage, and fiber splitting damage type appeared on the front face while fiber breakage and fiber splitting were developed at both the edge of the back face, in the case of S composite sample.

## 4. Conclusions

Tensile strength after the impact of original and repaired multi-impacted composite samples was examined and compared. Moreover, tensile load of the impacted original and repaired samples compared with non-impacted original and scarf-repaired samples; the influence of the low-velocity impact to the original and repair samples was investigated. The result identified the multi-impacts influenced both low-velocity impact and post-tensile results. The structural and residual integrity of the impacted samples were determined through the tensile test. The key point of doing this research was to investigate the multi-impact response to repaired/original composite samples. Current research leads to the following prominent conclusions:

- Time-contact force, force-displacement, and energy-time curve of the composite samples under low-velocity multiple impacts were observed, and it was concluded that the peak force was found highest and time duration was minimal for the first impacted original and repaired composites.
- Both types of composite samples at the first impact demonstrated highest peak force value, least time duration, least maximum displacement, least absorbed energy, minimal damage area, and smallest damage depth. Moreover, these all change with the number of impacts.
- At the fourth impact, both composites presented lowest peak force, maximum time duration, highest displacement, maximum absorbed energy, maximum damage area and highest value of damage depth.
- From visual inspection, it was found that impacted samples attained lesser damage on the front face than the backface.
- A direct relationship was established between absorbed energy and damage. The composite samples absorbed more energy and showed up more damage. In the case of repaired sample, the absorbed energy per impact was higher than the original sample.
- Samples without any impact showed better tensile results compared to impacted samples. The residual strength of the original sample was more than the repaired multi-impacted samples. Moreover, the first impacted sample contained the highest tensile load, whereas the fourth multi-impacted samples showed lowest tensile load-carrying capacity.
- Tensile tests after impact results showed that residual strength of the repaired sample was less than the original sample and reduced with number of impacts. The composite samples that demonstrated highest peak force among all during the impact test obtained more tensile strength. The post-tensile response analysis revealed that those composite samples which procured least absorbed energy/damage dent during impact test showed the highest ultimate load in post-tensile test.

## Figures and Tables

**Table 1:** Grouping of GFRP repaired and original composite samples.

GFRP Samples	Sample Group	Impact Times	Sample Code (Type)	Sample Number
Original samples	Group 1	1st	A	3
		2nd	B	3
		3rd	C	3
		4th	D	3
Repaired samples	Group 2	1st	P	3
		2nd	Q	3
		3rd	R	3
		4th	S	3

**Table 2:** The result of GFRP laminates performance under low-velocity impact test.

Impact No.	Maximum Force (N)		Maximum Displacement (mm)		Time Duration (ms)		Absorbed Energy (J)	
	Original	Repaired	Original	Repaired	Original	Repaired	Original	Repaired
**1st**	5749.091	5016.084	3.018	3.147	8.412	10.367	6.631	8.785
**2nd**	5525.032	4862.772	3.106	3.170	8.881	10.401	6.996	9.145
**3rd**	5226.412	4633.833	3.143	3.200	9.240	10.756	7.484	9.541
**4th**	4993.264	4513.674	3.245	3.289	9.322	10.834	7.849	9.901

**Table 3:** GFRP laminates performance tensile after impact test.

Impact No.	Failure Load (KN)		Percentage Load Difference with Respect to Original Samples (%)
	Original	Repaired	
Without impact	229.394	186.357	18.762
First impact	201.080	169.715	19.563
Second impact	198.304	161.403	18.602
Third impact	186.998	154.179	17.550
Fourth impact	183.768	152.378	17.081
